# BRIDGE: an interactive application for multi-omics data analysis, visualization and integration

**DOI:** 10.1093/bioinformatics/btag558

**Published:** 2026-07-27

**Authors:** David Márquez-Oller, Andrea Pauli, Jörg Fallmann

**Affiliations:** Research Institute of Molecular Pathology (IMP), Vienna BioCenter (VBC), Wien 1030, Austria; Research Institute of Molecular Pathology (IMP), Vienna BioCenter (VBC), Wien 1030, Austria; Research Institute of Molecular Pathology (IMP), Vienna BioCenter (VBC), Wien 1030, Austria

## Abstract

**Summary:**

BRIDGE is a Shiny-based application that provides an accessible, modular platform for individual and integrative multi-omics analysis. Using an independent SQLite database backend, it offers a local, private, and user-friendly environment that requires no prior computational expertise. The application supports proteomics, phospho-proteomics, and RNA-seq analyses through a comprehensive suite of visualization and analytical modules, together with an integrated multi-omics analysis pipeline. Built-in caching and asynchronous processing improve responsiveness, enabling efficient exploration, analysis, and visualization of multi-omics datasets on moderate hardware.

**Availability and implementation:**

BRIDGE is implemented in R using Shiny and is freely available as a Docker container at https://ghcr.io/paulilab/bridge. A public demonstration server with example datasets is available at https://bridge.imp.ac.at. Code and datasets are also available at https://github.com/paulilab/BRIDGE and under DOI: https://doi.org/10.5281/zenodo.20215824.

## 1 Introduction

Decreasing costs of high-throughput omics technologies enable rapid and widespread adoption of the latter in a growing number of research groups. However, the resultant stark increase in data availability has not been met with a matched development of easy-to-use analysis tools. Therefore, data analysis and integration remain a challenge. We address this challenge in our shiny application BRIDGE, which enables wet-lab scientists to analyze, visualize and integrate their own and published multi-omics data without extensive prior computational knowledge.

Existing applications like ROGUE (Farrel *et al.* 2023) or the shiny app associated with the DEP2 package (Feng *et al.* 2023) provide user-friendly interfaces for analyzing and visualizing data. However, these two applications lack an integration pipeline for different types of datasets. The latter has become crucial for investigating complex biological processes on multiple levels, which often requires multi-omics approaches where integration becomes a crucial bottleneck in data analysis. The recently published application OmNI (Potter *et al.* 2026) addresses this problem by presenting a fully-fledged, interactive analysis pipeline for in-depth multi-omics data integration and visualization. While OmNI is a powerful tool, it requires significant coding skills and has limited accessibility for non-experts.

Here, we present BRIDGE, which addresses the growing need for a built-in, lightweight integration pipeline for different types of datasets (proteomics, phospho-proteomics and RNAseq). BRIDGE bridges the gap between accessibility, interactivity, comprehensiveness, responsiveness and light-weightedness. It integrates (i) modular analysis methods utilizing wrapper functions provided by established packages (e.g. DEP2, clusterProfiler (Yu *et al.* 2012)) together with (ii) state-of-the art visualization tools (e.g. ComplexHeatmap (Gu 2022), EnhancedVolcano (Blighe 2018) and interactive PCA), (iii) responsive data processing (tidyverse (Wickham *et al.* 2019) and tidySummarizedExperiment (Hutchison *et al.* 2024)) and (iv) a caching database with a shareable, but private, data back-end, which sets it apart from other existing apps.

BRIDGE enables differential expression analysis (DEA), gene ontology (GO) and pathway enrichment, together with interactive visualizations in the form of heatmaps, volcano and PCA plots. Additionally, it provides an easy-to-use and lightweight integration pipeline to join two or more datasets of the same or different data types, which facilitates comprehensive analyses of even more complex and multi-type data. Integration is based on the intersection of either raw or processed datasets, which enables consolidating and visualizing data and results, aiding in biological interpretation and easy retrieval for further downstream analysis. The SQLite (https://www.sqlite.org/index.html) backend provides high-performance storage and fast retrieval of large datasets, while also enabling data sharing or protection independent of the application.

With BRIDGE, deposited on github (https://github.com/paulilab/BRIDGE) and Zenodo (https://doi.org/10.5281/zenodo.20215824), we provide a free, accessible and user-friendly application for data exploration, visualization and analysis. It is open-access, interoperable, follows best practice data formats and standards and is reusable via modularization and containerization in line with FAIR principles (https://www.go-fair.org/fair-principles/). Extensive documentation (https://bridge-paulilab.readthedocs.io/en/latest/), enables rapid analyses and lightweight integration of multi-omics (proteomics, phosphoproteomics and RNA-seq) data.

## 2 BRIDGE

A user- or group-specific, local SQLite database, generated from raw and/or processed data forms the backend of BRIDGE. We provide python scripts and a stand-alone shiny app (app_db_builder.R) to convert CSV or TSV files, e.g. directly from next-generation sequencing (NGS) or mass spectrometry (MS) service providers or exported from Excel sheets into a standardized SQL format. Adherence to a set of rules, explained in detail in the documentation online, enables integration and analysis of diverse datasets via stable identifiers, e.g. ENSEMBL (Dyer *et al.* 2025) IDs. This design enables private storage as well as straightforward data sharing with collaborators via independent database files. The backend also functions as a cache for rapid retrieval of processed results and plots, accelerating data exploration in successive runs.

The core component of the system is a Shiny-based application developed in R (Chang and Borges Ribeiro 2021, Chang *et al.* 2024, Perrier *et al.* 2025). The user interface (UI) provides download-ready results, tables and visualizations for raw and processed data exploration, including differential expression displays such as heatmaps and volcano plots.

Standard data analysis for proteomics, phospho-proteomics and RNA-seq data is provided via an integrated pipeline based on DEP2. Data types are automatically recognized via a standardized naming scheme, and analysis starts with data import. As soon as the pipeline has processed the data, users can investigate and download results via above mentioned tabular or graphical interfaces.

Implemented standard analysis consists of simple design models (∼ 0 + condition), where conditions are based on columns selected by the user on application startup. If more than two conditions are available, we employ an all vs all approach with pairwise comparisons. Each selection of columns by the user is stored as a uniquely identifiable object in the database, which is then used to quickly retrieve results on subsequent loading of the same columns from the same table.

Users can download processed SummarizedExperiment RDS files from the app for manual inspection, further analysis or re-upload to a new database. The processing pipeline running in the background is streamlined to provide a best-practice implementation of RNAseq/MassSpec data analysis without requiring additional settings from the user. In case a more complex design is needed, e.g. batch effects are visible in PCA plots and batch correction becomes necessary, the user can download the processed tables or RDS objects, manually process them with tools like DEP2 or DESeq2 (Love *et al.* 2014) and use those to create a database for BRIDGE. Such a flexible implementation strategy serves both beginners and experts in data exploration and analyses: it allows beginners to analyze their data with limited to no prior expertise, while more experienced users can process their data in a customized manner while still benefiting from the visualization and integration features provided. Furthermore, upload of processed SummarizedExperiments objects together with raw data tables facilitates the transfer of analysis results from bioinformaticians to wet-lab biologists or collaborators, who can then utilize BRIDGE for further exploration of the results. The accompanying Python scripts and R app guide the user through a simple, text-based interface or UI to select columns containing data, identifiers and metadata from input files and optionally ask for paths to pre-existing R objects, which will then be cached in the database similar to in-app generated objects.

A key feature of BRIDGE is its ability to integrate and thereby simultaneously explore multiple datasets across omics realms, which is essential for addressing complex biological questions that span multiple molecular layers. This feature is intentionally kept lightweight and based on intersection of feature IDs or names to provide-ready-to-download tables in a streamlined format. Thus, results of the integration pipeline can be used for further processing with dedicated integration techniques, see e.g. Baião *et al.* (2025) for an overview, which all profit from curated input.

BRIDGEs modular architecture separates UI components, server logic, and helper functions, facilitating maintenance and extension also to additional omics data types or different techniques of integration. All source code is available under the Apache License 2.0 at https://github.com/paulilab/BRIDGE, which also provides detailed documentation, extensive help, and information on the implemented packages. For convenience and easy deployment, we provide a docker container at https://ghcr.io/paulilab/bridge, which will be regularly updated on app changes and development, as well as a showcase server hosted at https://bridge.imp.ac.at to enable users to first test the application to find out whether it fits their needs.

BRIDGE is compartmentalized into a raw analysis part that enables users to interactively examine individual datasets in table format and via heatmaps and timeline plots. Optional data transformation of counts to commonly used units including log2, TPM, CPM, and FPKM facilitates quality control (QC) and identification of global patterns or candidate features in each dataset. The processed data analysis extends these capabilities by fully automatized differential expression analysis, interactive volcano plots, and heatmaps. Functional interpretation is supported by GO term and pathway enrichment analyses and an interactive principle component analysis (PCA) module, which offers a global view of sample relationships, data quality and principle component loadings.

**Figure 1:**
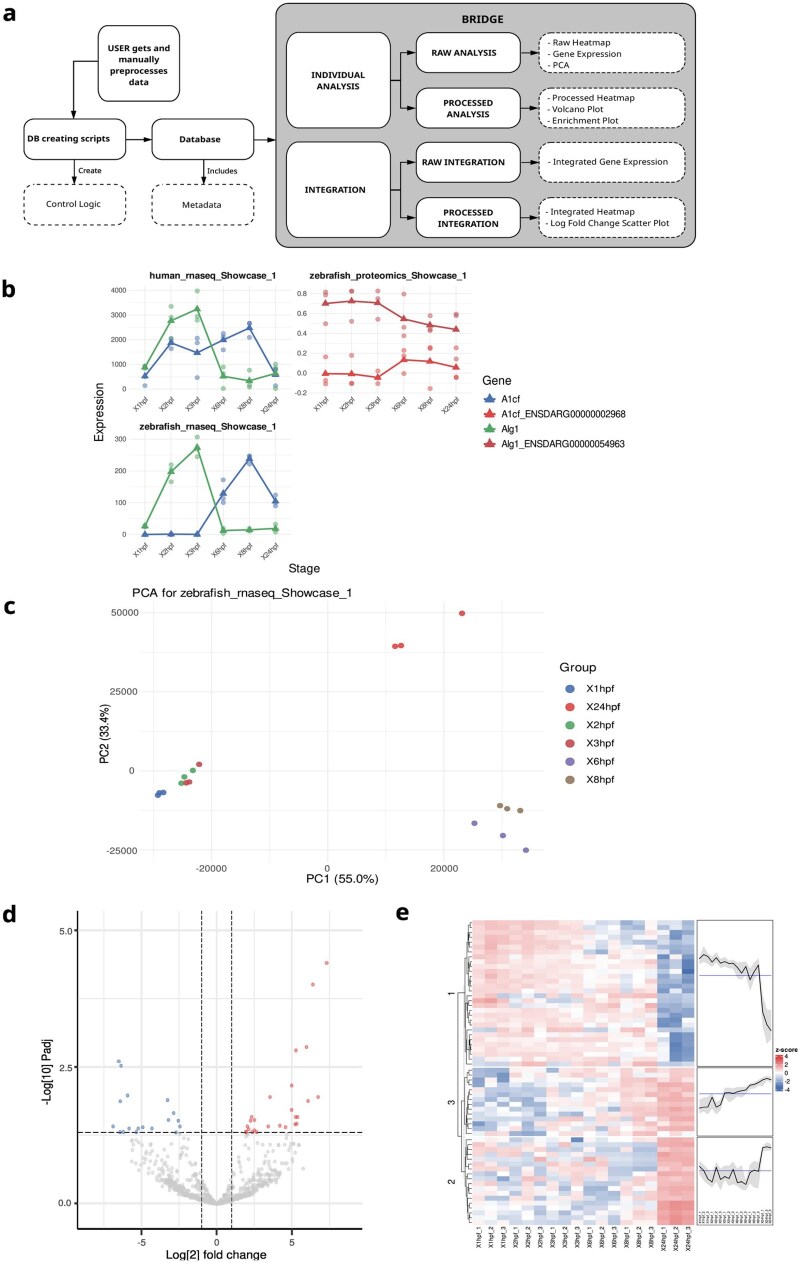
(a) BRIDGE workflow: Diagram depicting the workflow and visualization functionalities of BRIDGE from data retrieval to individual and integrated analysis. (b–e) Expression profiles, PCA, Volcano plot and Heatmap visualizations obtained from the app with mock data.

In addition to facilitating the analysis of individual data set modalities (proteomics, phosphoproteomics and RNA-seq), BRIDGE enables lightweight integration of different omics techniques via two separate integration pipelines: The raw-integration module merges datasets through shared identifier columns, enabling joint visualization, filtering and QC of expression profiles. The processed-integration module identifies overlaps among significant differentially expressed features across datasets, highlighting concordant or divergent regulation across, for example, RNA and protein levels. Interactive visualizations, including heatmaps and comparative scatter plots, enable identification of conserved trends and dataset-specific dynamics (see Fig. 1).

BRIDGE’s modular, open-source architecture enables straightforward extension to additional omics types and analysis modules, making it adaptable to diverse research contexts and attractive for collaborative research settings.

## 3 Discussion

BRIDGE is an interactive application for analysis, visualization and integration of multi-omics data independently of the species from which the data was derived. It currently supports RNA-seq, proteomics, and phospho-proteomics data, combining a local SQLite database backend with a shiny-based graphical interface. The backend ensures secure storage of user data and annotations, while the frontend offers a modular interface for exploring and analyzing both raw and processed data.

A dedicated integration framework supports integration of both raw and processed data across multi-omics modalities. BRIDGE incorporates performance optimizations, such as a caching system and asynchronous processing, that scale well with input size and analysis demands.

A test server (https://bridge.imp.ac.at) with four simulated datasets, one of each type of currently implemented omics techniques from zebrafish plus an RNAseq dataset from human, together with corresponding annotations enables users to explore the application’s functionality without any setup requirements. A docker container is available for easy deployment at https://ghcr.io/paulilab/bridge:latest.

BRIDGE provides an accessible, modular platform for single-omics and integrative multi-omics analysis through a user-friendly GUI that requires minimal to no computational background. Its modular architecture facilitates extension to new omics types and analytical functions. This establishes BRIDGE as a lightweight yet powerful and reliable platform that strengthens the integrative multi-omics ecosystem and allows researchers, with or without coding expertise, to explore, analyze, and share their data effectively.

## Data Availability

Code and datasets are available at https://github.com/paulilab/BRIDGE and under https://doi.org/10.5281/zenodo.20215824.
